# TFPI-2 is a putative tumor suppressor gene frequently inactivated by promoter hypermethylation in nasopharyngeal carcinoma

**DOI:** 10.1186/1471-2407-10-617

**Published:** 2010-11-09

**Authors:** Shumin Wang, Xue Xiao, Xiaoying Zhou, Tingting Huang, Chunping Du, Nana Yu, Yingxi Mo, Longde Lin, Jinyan Zhang, Ning Ma, Mariko Murata, Guangwu Huang, Zhe Zhang

**Affiliations:** 1 Department of Otolaryngology Head and Neck Surgery, First Affiliated Hospital of Guangxi Medical University, Nanning, PR China; 2 Department of Environmental and Molecular Medicine, Mie University Graduate School of Medicine, Japan; 3 Faculty of Health Science, Suzuka University of Medical Science, Japan

## Abstract

**Background:**

Epigenetic silencing of tumor suppressor genes play important roles in NPC tumorgenesis. Tissue factor pathway inhibitor-2 (TFPI-2), is a protease inhibitor. Recently, *TFPI-2 *was suggested to be a tumor suppressor gene involved in tumorigenesis and metastasis in some cancers. In this study, we investigated whether *TFPI-2 *was inactivated epigenetically in nasopharyngeal carcinoma (NPC).

**Methods:**

Transcriptional expression levels of *TFPI-2 *was evaluated by RT-PCR. Methylation status were investigated by methylation specific PCR and bisulfate genomic sequencing. The role of *TFPI-2 *as a tumor suppressor gene in NPC was addressed by re-introducing *TFPI-2 *expression into the NPC cell line CNE2.

**Results:**

*TFPI-2 *mRNA transcription was inactivated in NPC cell lines. *TFPI-2 *was aberrantly methylated in 66.7% (4/6) NPC cell lines and 88.6% (62/70) of NPC primary tumors, but not in normal nasopharyngeal epithelia. *TFPI-2 *expression could be restored in NPC cells after demethylation treatment. Ectopic expression of TFPI-2 in NPC cells induced apoptosis and inhibited cell proliferation, colony formation and cell migration.

**Conclusions:**

Epigenetic inactivation of *TFPI-2 *by promoter hypermethylation is a frequent and tumor specific event in NPC. *TFPI-2 *might be considering as a putative tumor suppressor gene in NPC.

## Background

Nasopharyngeal carcinoma (NPC) is a rare disease in most parts of the world but is one of the most prevalent malignasnt tumors and the leading cause of death among all head and neck cancers in Southern China[1]. Although the major etiological factors, including genetic susceptibility, environmental carcinogens and Epstein-Barr virus infection, are well established, the complicated molecular basis of NPC development and progression remains unclear[1]. In addition to genetic alterations, epigenetic inactivation of tumor suppressor genes (TSGs) by promoter hypermethylation has been increasingly recognized as a key event for nasopharyngeal carcinogenesis[1-4]. Compared with the incidence of DNA mutation and deletion, that of aberrant DNA methylation of TSGs is high in NPC, which suggests a deep involvement of aberrant DNA methylation in this rare cancer[1].

Besides having a role as a diagnostic or prognostic bio-marker, hypermethylated DNA can also be used to reveal novel TSGs[5]. Identifying aberrant methylated genes may provide better understanding of the pathogenesis of NPC, thereby paving the way for the development of novel tumor markers and therapeutic targets. To discover novel TSGs in NPC, we conducted a genome-wide screening for genes downregulated by promoter hypermethylation. Analysis of an expression microarray with RNA from 2 NPC cell lines before and after treatment with a demethylating agent 5-aza-2'-deoxycytidine (5-aza-dC) revealed the transcriptional levels of *TFPI-2 *in both cell lines significantly upregulated after demethylation(unpublished data). Thus, *TFPI-2 *might be a target gene with expression suppressed by promoter hypermethylation in NPC.

The human *TFPI-2 *gene is located on chromosome 7q22 and encodes a 32-kDa Kunitz-type serine protease inhibitor that negatively regulates the enzymatic activity of trypsin, plasmin, and VIIa-tissue factor complex[6-8]. *TFPI-2 *was found to be abundantly expressed in various normal human tissues, including seminal vesicles, colon, stomach, brain, pancreas, oesophagus and liver[9-13]. Several studies have provided suggestive evidence that *TFPI-2 *is inactivated or absent during tumor progression. In addition, expression of *TFPI-2 *decreases with an increasing degree of malignancy[10]. Besides gene locus deletion and aberrant splicing, the mechanism responsible for *TFPI-2 *down-regulation in tumor cells has been majorly attributed to promoter hypermethylation[14-16]. The promoter region of *TFPI-2 *contains a CpG island spanning exon 1 and the 3 transcription initiation sites[17]. This region harbors sites for several transcription factors, including SP1, activiting enhancer-binding protein-1 (AP-1), NF-kB and NF-kB-like site, and lymphoid transcription factor-1 (Lyf-1)[18]. Transcriptional silencing by promoter hypermethylation of *TFPI-2 *was observed in some human cancers[9,19,20]. Recent studies also suggest *TFPI-2 *may function in the maintenance of the stability of the tumor environment and inhibit the growth of neoplasms, thus act as a candidate TSG with important roles in carcinogenesis and metastasis in human cancers[9,12].

In this study, we evaluated the transcriptional levels and methylation status of *TFPI-2 *in NPC cell lines and analyzed the methylation status of *TFPI-2 *in a set of NPC primary tumor biopsies. We further addressed the TSG properties of *TFPI-2 *in NPC by a series of functional experiments. Several lines of evidence support our hypothesis that *TFPI-2 *is epigenetically inactivated in NPC by promoter hypermethylation and plays a role as a TSG in NPC tumorigenesis.

## Methods

### NPC cell lines, primary tumor biopsies and normal nasopharyngeal epithelia (NNE)

Six NPC cell lines (CNE1, CNE2, TW03, C666-1, HNE1, and HONE1) were maintained at 37°C in the appropriate medium. The study was approved by the ethics committee of Guangxi Medical University. In total, 70 NPC primary tumor biopsies were collected from Department of Otolaryngology Head and Neck Surgery, First Affiliated Hospital of Guangxi Medical University (Nanning, China), with informed consent from donors, as previously described[2,3]. Diagnoses were established by experienced pathologists according to the World Health Organization (WHO) classification. We included 12 normal nasopharyngeal epithelial tissues obtained by tonsillectomy as normal controls. Biopsy samples were stored in liquid nitrogen before DNA or RNA extraction.

### Semi-quantitative RT-PCR

Total RNA of NPC cell lines and normal NNE was isolated with TRIzol reagent (Invitrogen, USA). First-strand cDNA was synthesized with M-MLV reverse transcriptase (Promega, USA) according to the manufacturer's instructions. Two micrograms of total RNA was used for each reaction. Primer sequences for *TFPI-2 *cDNA were designed according to Norihiro *et al*, generating a 209-bp PCR product: TFPI-2-RT-forward: CCAGATGAAGCTACTTGTATG and TFPI-2-RT-reverse: GCACATGCACGTTTGCAATC [21]. PCR was carried out in a total volume of 20 μl. The PCR mixture contained 250 pmol of each primer, 250 pmol deoxynucleoside triphosphate, 1 × PCR buffer, one unit of ExTaq HS polymerase (Takara, Tokyo), and 2 μl cDNA. Glyceraldehyde-3-phosphate dehydrogenase (*GAPDH*) was amplified from the same cDNA sample as the internal control. The primer sequences for *GAPDH *cDNA were: GAPDH-RT-forward: AAGCTCACTGGCATGGCCTT, and GAPDH-RT-reverse: CTCTCTTCCTCTTGTGCTCTTG, generating a 375-bp PCR product. PCR conditions were 94°C for 30 s, 58°C for 30 s (*TFPI-2*) or 60°C for 30 s (*GAPDH*), and 72°C for 30 s, 33 cycles for the *TFPI-2 *gene and 24 cycles for the *GAPDH *gene. These cycle numbers fell into the exponential range of PCR amplification. The amplified PCR products were then identified on 2% agarose gels. Images of ethidium bromide-stained agarose gels were acquired with a CCD camera (Bio-Rad, USA), and semi-quantitative analysis involved use of the Quantity-one software, v4.4.0 (Bio-Rad, USA).

### Sodium bisulphite modification of genomic DNA

High-molecular-weight genomic DNA was extracted from cell lines and biopsies by a conventional phenol/chloroform method. The sodium bisulphite modification procedure was as described [22] with slight modification. In brief, 600 ng of genomic DNA was denatured in 3 M NaOH for 15 min at 37°C, then mixed with 2 volumes of 2% low-melting-point agarose. Agarose/DNA mixtures were then pipetted into chilled mineral oil to form agarose beads. Aliquots of 200 μl of 5 M bisulphite solution (2.5 M sodium metabisulphite, 100 mM hydroquinone, both Sigma, USA) were added into each tube containing a single bead. The bisulphite reaction was then carried out by incubating the reaction mixture for 4 h at 50°C in the dark. Treatments were stopped by equilibration against 1 ml of TE buffer, followed by desulphonation in 500 μl of 0.2 M NaOH. Finally, the beads were washed with 1 ml of TE buffer and directly used for PCR.

### Methylation-specific PCR

The methylation status of the *TFPI-2 *promoter region was determined by methylation-specific PCR. Primers distinguishing unmethylated (U) and methylated (M) alleles were designed to amplify the sequence of *TFPI-2 *from - 44 to + 60 bp relative to the transcription start point[21]: TFPI-2-M-forward: TTTCGTATAAAGCGGGTATTC; TFPI-2-M-reverse: ACGACCCGCTAAACAAAACG; TFPI-2-U-forward: GGATGTTTGTTTTGTATAAAGTG; TFPI-2-U-reverse: AAACATCCAAAAAAACACCTAAC. Each PCR reaction contained 20 ng of sodium bisulphite-modified DNA, 250 pmol of each primer, 250 pmol deoxynucleoside triphosphate, 1 × PCR buffer, and one unit of ExTaq HS polymerase (Takara, Tokyo) in a final reaction volume of 20 μl. Cycling conditions were initial denaturation at 95°C for 3 min, 40 cycles of 94°C for 30 s, 54°C (M) or 60°C (U) for 30 s, and 72°C for 30 s. For each set of methylation-specific PCR reactions, *in vitro*-methylated genomic DNA treated with sodium bisulphite served as a positive methylation control. A water blank control was also included. PCR products were separated on 2% agarose gels, stained with ethidium bromide and visualized under UV illumination. For cases with borderline results, PCR analyses were repeated.

### Bisulphite genomic sequencing

Sodium bisulphite-modified DNA was subjected to PCR with primers designed to amplify nucleotides from - 178 to + 166 bp relative to the transcription start point of the *TFPI-2 *gene. Primers sequences were designed according to previous publication by Norihiro *et al. *[21] TFPI-2-BISQ-forward: AGGTAGGTTTAATTTTTTAATTTG and TFPI-2-BISQ-reverse: TACCTATTAACTCCTAAACAAC. PCR was carried out in a total volume of 20 μl, containing 20 ng of sodium bisulphite-modified DNA as template, 250 pmol of each primer, 250 pmol of deoxynucleoside triphosphate, 1× PCR buffer, and one unit of ExTaq HS polymerase (Takara, Tokyo). Cycling conditions were as follows: initial denaturation at 95°C for 3 min, 40 cycles of 94°C for 30 s, 58°C for 30 s, and 72°C for 30 s, and a final extension at 72°C for 5 min. PCR products were then gel-purified and cloned with use of the pMD18-T Vector (Takara, Tokyo) and JM109-competent *E. coli *cells. Colonies were grown on agar plates, and 5 colonies of each sample were randomly selected. Plasmids were then isolated and purified. Sequencing were carried out using the BigDye terminator cycle sequencing kit 3.0 (Applied Biosystems, USA) on an ABI 3100 sequencer according to the manufacturer's guidelines.

### 5-aza-dC treatment

Three NPC cell lines (CNE1, CNE2 and C666-1) were treated with the methyltransferase inhibitor 5-aza-dC. An amount of 2 × 10^5 ^cells were seeded on 6-well plates and incubated for 96 h with 10 μM 5-aza-dC. The medium containing the drug was replaced every 24 h. RNA was isolated, and subsequent semi-quantitative RT-PCR was performed as described previously.

### Vector construction and transfection

The full-length cDNA sequence of *TFPI-2 *was from Origene (USA) and subcloned into the pCMV-Tag3A mammalian expression vector (Stratagene, USA). Lipofectamine 2000 (Invitrogen, USA) was used to transfect the *TFPI-2-*containing plasmids and empty vectors into CNE2 cells, which lack *TFPI-2 *expression. Stable clones of *TFPI-2 *(CNE2-*TFPI-2*) or empty vector (CNE2 empty vector) were obtained by G418 selection (400 μg/ml) for 2 weeks. The expression of exogenous *TFPI-2 *was confirmed by RT-PCR.

### Colony formation assay

An amount of 10^5 ^CNE2 cells was plated in 24-well plates 24 h before transfection. Lipofectamine 2000 was used for transfection according to the manufacturer's protocol. Cells were transfected with 2 μg pCMV-Tag3A-*TFPI-2 *plasmid or empty vector. Transfected cells were stripped and plated on 60-mm cell culture dishes 48 h after transfection. After selection by 400 μg/ml G418 for 2 weeks, Giemsa-stained colonies were photographed and counted by use of Quantitione software, v4.4.0 (Bio-Rad, USA).

### Cell proliferation assay

The growth curves of CNE2 cells stably transfected with *TFPI-2 *gene (CNE2-*TFPI-2*) or empty vector (CNE2-empty vector) were monitored by cell counting. An amount of 10^4 ^cells of parent CNE2 cells, CNE2-*TFPI-2 *and CNE2-empty vector were seeded into 6-well plates. Cells were trypsinized and stained with a 0.4% trypan blue solution every 24 h. Dye-exclusive cells were counted by use of a hemocytometer.

### Migration study

Cell migration was assessed by *in vitro *wound-healing assay. Stable transfectants of CNE2, CNE2-*TFPI-2 *and CNE2-empty vector were cultured on 35-mm dishes for 24-48 h until they reached confluence. A linear wound track was made by use of a sterile tip. Wounds were marked and scored in each dish. Plates were then washed twice with culture medium. Cells migrating into the wound were photographed under a phase-contrast microscope after 24 h incubation after wounding.

### Annexin V-FITC/PI double-labeled flow cytometry

To determine apoptosis, the expression of Annexin V-FITC and exclusion of propidium iodide (PI) was detected by double-labeled flow cytometry. Cells transfected with empty vector and *TFPI-2 *were collected and washed with PBS 24 h after transfection, then resuspended in 100 μL binding buffer. Samples were incubated with 5 μL Annexin V-FITC in the dark for 10 min at 4°C, then the volume was adjusted to 500 μL with binding buffer. PI (5 μL) was added, and samples were incubated for another 10 min at 4°C. Fluorescence was measured with a flow cytometer (BD FACSCalibur, USA).

### Statistical analysis

SPSS v11.5 (SPSS Inc., Chicago, IL) was used for statistical analysis. Association between methylated sample data and clinical pathological features of NPC patients were analyzed by Pearson chi-square test or Fisher's exact test. A *p *< 0.05 was considered statistically significant.

## Results

### *TFPI-2 *mRNA expression is frequently absent in NPC cell lines

**Figure 1:**
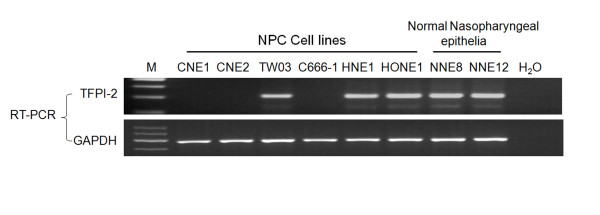
**RT-PCR analysis of mRNA expression of TFPI-2 in NPC cell lines and normal nasopharyngeal epithelia (NNE) samples**. The data are representative of 2 independent experiments. Glyceraldehyde-3-phosphate dehydrogenase (*GAPDH*) and water were used as internal and blank controls, respectively.

*TFPI-2 *mRNA expression was evaluated in 6 NPC cell lines (CNE1, CNE2, TW03, C666-1, HNE1 and HONE1) and in 12 biopsies of normal NNE by semi-quantitative RT-PCR. *TFPI-2 *mRNA was detected in all NNE (Figure 1, NNE8 and NNE12 as samples). Of the 6 NPC cell lines tested, 3 lines (CNE1, CNE2 and C666-1) showed complete silencing of *TFPI-2 *expression, and decreased expression was detected in TW03, HNE1 and HONE1 cell lines (Figure 1).

### *TFPI-2 *promoter is hypermethylated in NPC cell lines and primary NPC tumors

**Figure 2:**
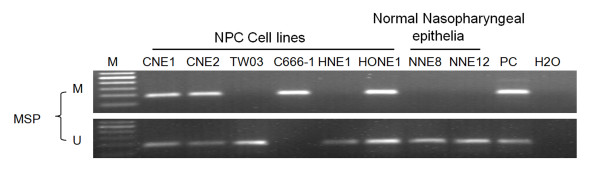
**Methylation status of the TFPI-2 promoter region in NPC cell lines and normal nasopharyngeal epithelia (NNE)**. The data are representative of 2 independent experiments. *In vitro*-methylated DNA was used as a methylation-positive control and DNA from normal lymphocytes was used as an unmethylated-positive control. Water was included as a blank control. MSP: methylation-specific PCR; U: unmethylated alleles; M: methylated alleles. PC: positive control.

**Figure 3:**
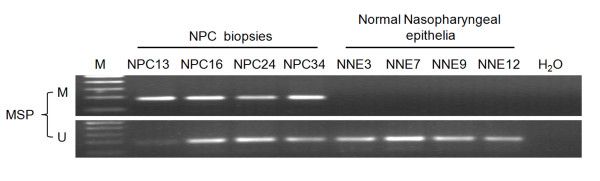
**Methylation-specific PCR analysis of the TFPI-2 promoter region in NPC primary tumors and normal nasopharyngeal epithelia (NNE)**. Four NPC primary tumors (NPC 13, 16, 24 and 34) and 4 NNE (NNE3, 7, 9 and 12) are shown as examples. U: unmethylated alleles; M: methylated alleles. The data are representative of 2 independent experiments.

The methylation status of the *TFPI-2 *promoter in NPC cell lines was detected by methylation-specific PCR assay. The *TFPI-2 *promoter was hypermethylated in 4 of 6 NPC cell lines (CNE1, CNE2, C666-1 and HONE1); only TW03 and HNE1 cells were unmethylated (Figure 2). All 12 normal nasopharyngeal epithelial tissues showed unmethylated *TFPI-2 *promoters. We further evaluated the methylation status of the *TFPI-2 *promoter in 70 NPC primary tumors. Promoter hypermethylation was detected in 88.6% (62/70) of the NPC primary tumors but in none of the 12 NNE. Representative samples are shown in Figure 3. Unmethylated amplicons were found in some but not all of the NPC biopsy samples likely because of the existence of non-malignant cells such as stromal cells in a fraction of the samples.

### CpG sites in the TFPI-2 promoter are heavily methylated in NPC cell lines and NPC biopsies

**Figure 4:**
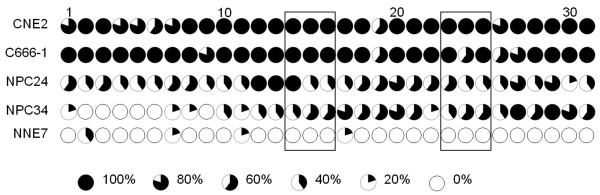
**Bisulphite genomic sequencing of NPC cells, tumor biopsies and NNEs**. Bisulphite genomic sequencing of the methylation status of the 31 CpG sites within the *TFPI-2 *promoter in 2 NPC cell lines (CNE2 and C666-1), 2 NPC biopsies (NPC24 and NPC34) and 1NNE biopsy (NNE7). Five randomly selected clones were sequenced for each sample. Open and filled circles represent unmethylated and methylated CpG sites, respectively. Circles were partially filled according to the percentage of methylation of the CpG site. The frames show the CpG pairs covered by methylation-specific PCR primers.

To detect the methylation status of individual CpG sites in the NPC samples relative to the normal control biopsies, the detailed methylation status of the *TFPI-2 *promoter region - 178 to + 166 bp relative to the translation start site of the *TFPI-2 *gene was determined by bisulphite genomic sequencing in 2 NPC cell lines (CNE2 and C666-1), 2 NPC biopsies (NPC24 and NPC34) and 1 NNE sample (NNE7). The relevant 344-bp *TFPI-2 *promoter region contained 31 CpG sites. For each sample shown in Figure 4, the sequence of 5 representative clones is shown. All 31 CpG sites were heavily methylated in cell lines CNE2 and C666-1, in which *TFPI-2 *expression was silenced (Figure 4). Dense methylation of CpG dinucleotides was also observed in the 2 NPC biopsies tested. The presence of some unmethylated clones in the CNE2 and C666-1 cell lines may reflect heterogeneity in the original cell lines. The NNE samples were completely free of methylation. Bisulphite genomic sequencing also demonstrated that our bisulphite conversion of genomic DNA was complete and thus supports the reliability of our methylation-specific PCR results.

### Transcription of *TFPI-2 *can be restored by 5-aza-dC treatment

**Figure 5:**
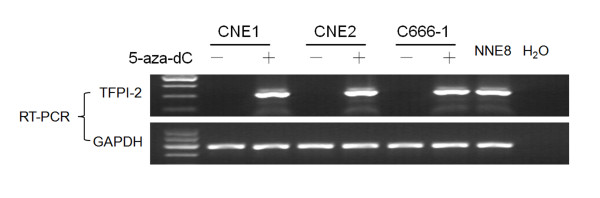
**Treatment with 5-aza-dC restores *TFPI-2 *expression in 3 NPC cell lines**. *TFPI-2 *mRNA expression level was evaluated by RT-PCR. *GAPDH *was amplified as an internal control. NNE8 was used as positive control. A water blank control was also included.

To confirm that methylation of the *TFPI-2 *gene is directly responsible for the loss of *TFPI-2 *transcription, the *TFPI-2*-silenced NPC cell lines (CNE1, CNE2 and C666-1) were treated with the demethylating agent 5-aza-dC for 4 days. Transcriptional expression was restored in all 3 cell lines (Figure 5).

### Clinico-pathological significance of *TFPI-2 *promoter hypermethylation

Analysis of the association of *TFPI-2 *promoter hypermethylation and clinico-pathological parameters of NPC patients revealed no significant association of presence of methylated or unmethylated *TFPI-2 *promoters and age, sex, cancer staging, or pathological subtypes (Table 1).

**Table 1:** Associations between *TFPI-2 *promoter methylation and clinicopathological indices of NPC

		Promoter methylation status		
			
	Number of patients	Methylated	Unmethylated	Statistic significance
Age				
< 60	58	52(89.7%)	6	NS
≥ 60	12	10(83.3%)	2	
				
Gender				
Male	48	44(91.7%)	4	NS
Female	22	18(81.8%)	4	
				
Stage*				
I-II	28	24(85.7%)	4	NS
III-IV	42	38(90.5%)	4	
				
Histological subtype				
Keratinizing squamous cell carcinoma	8	6(75%)	2	NS
Non-keratinizing carcinoma	62	56(90.3%)	6	
				
Lymph node metastasis				
Presence	58	50(86.2%)	8	NS
Absence	12	12(100%)	0	

*: Staging according to International Union Against Cancer (UICC).

NS: not statistically significant

### *TFPI-2 *suppresses cell proliferation and colony formation and inhibits cell migration in NPC cells

**Figure 6:**
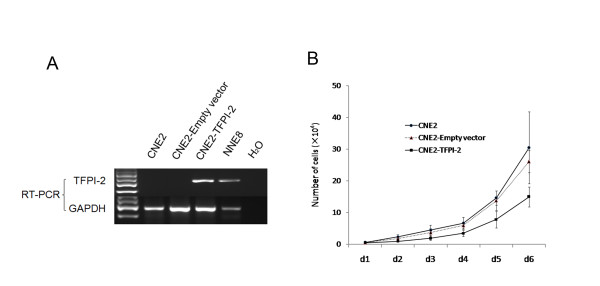
***TFPI-2 *inhibits NPC cell proliferation**. A: RT-PCR validation of stable transfectance of CNE2-TFPI-2 or CNE2-Empty vector. B: Proliferation curves of CNE2 cells, stable transfectants of CNE2-*TFPI-2 *and CNE2-empty vector. Cells were counted every 24 h for 6 days.

**Figure 7:**
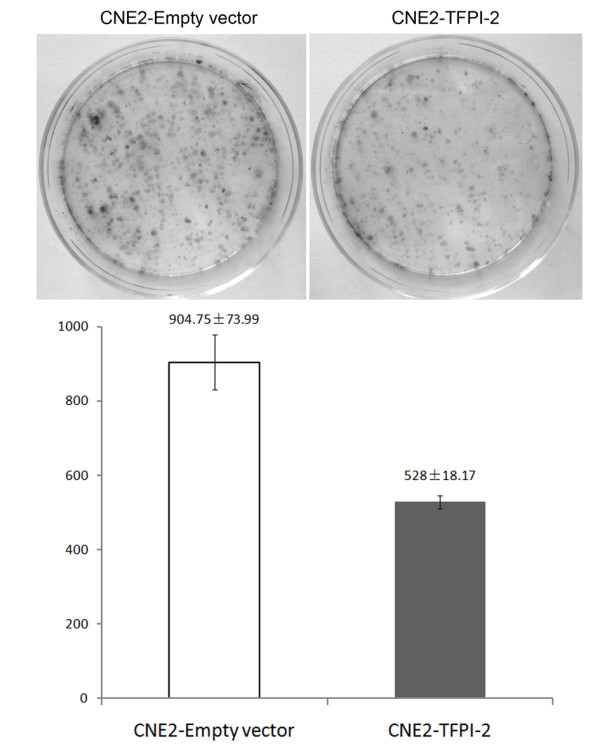
***TFPI-2 *inhibits colony formation in NPC cells**. Top: CNE2 cells were transfected with empty vector or *TFPI-2*-expressing plasmids and selected with G418 for 2 weeks. The experiment was done in triplicate and the error bars represent standard deviations (*p *< 0.05).

**Figure 8:**
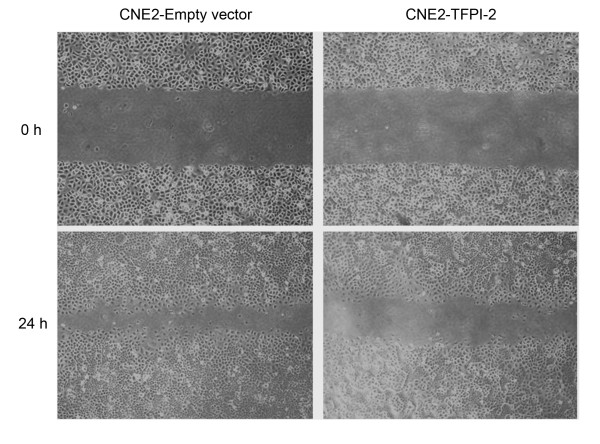
***TFPI-2 *inhibits cell mobility in NPC cells**. The cell motility of the CNE2-empty vector and CNE2-*TFPI-2 *was determined by wound healing assay. At 24 h after scratching, the CNE2-*TFPI-2 *cells spread significantly slower than did CNE2 empty vector cells. All experiments were performed in triplicate, and data from representative experiments are shown.

To assess whether *TFPI-2 *might possess properties as a tumor suppressor gene in NPC, we examined the effect of *TFPI-2 *on clonogenicity, cell proliferation, and cell mobility. The colony-formation efficiencies were evaluated by monolayer culture. The number of colonies formed by CNE2-*TFPI-2 *cells was less than that by CNE2-empty vector cells (*p *< 0.05) (Figure 7). This finding was further supported by cell proliferation assay. CNE2-*TFPI-2 *cells grew significantly slower than CNE2 parental cells and CNE2-empty vector cells (Figure 6). Wound healing assay was carried out to measure cell mobility by comparing the scratching healing efficiency of CNE2-*TFPI-2 *cells and CNE2 empty vector cells. The CNE2-*TFPI-2 *cells moved slower into the scratched areas than did CNE2-empty vector control cells, which suggests that ectopic expression of *TFPI-2 *inhibits the motility of NPC cells (Figure 8).

### *TFPI-2 *induces apoptosis in NPC cells

**Figure 9:**
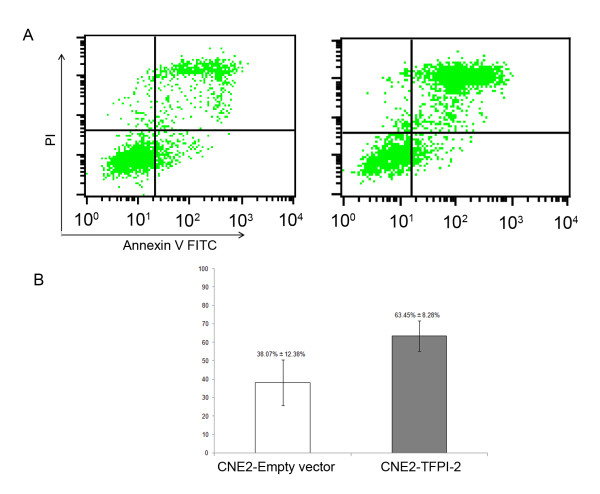
***TFPI-2 *induces apoptosis in NPC cells**. A: CNE2 cells were transfected with empty vector and *TFPI-2*, then stained with annexin V-FITC and PI and subjected to flow cytometry. Fluorescence dot blots of annexin V-positive (horizontal axis) and PI-positive (vertical axis) cells are shown. B: Cells that were positively stained by annexin V-FITC only (early apoptosis) and positive for both annexin V-FITC and PI (late apoptosis) were quantitated, and both subpopulations were considered as apoptotic cells. The bar graph shows the average in 5 experiments. Error bars show standard deviations.

The degree of early apoptosis was represented as a percentage of the annexin V-FITC-positive and PI-negative cells, and late apoptosis was quantitatively represented as a percentage of the annexin V-FITC-positive and PI-positive cells. The overall apoptosis rate was the sum of early and late apoptosis subpopulations. The apoptosis rate in CNE2-TFPI-2 cells (63.45% ± 8.28%) was significantly higher than that in the empty vector control (38.07% ± 12.38%) in 5 independent tests (Figure 9), which suggests that TFPI-2 induces apoptosis in NPC cells.

## Discussion

Previously, we analyzed expression microarray data before and after treatment with demethylating agent in 2 NPC cell lines and speculated that the *TFPI-2 *gene may be downregulated by promoter hypermethylation in NPC cells. TFPI-2, also known as placental protein 5 and matrix-associated serine protease inhibitor [7,23], is a novel serine protease inhibitor[7,24]. It was reported to be involved in tumorigenesis and metastasis in several types of cancers[24]. Suppression of *TFPI-2 *gene expression is frequently found in melanoma, liver and pancreatic cancer[9,25,26]. Downregulation of *TFPI-2 *mRNA and protein by promoter hypermethylation has been confirmed by RT-PCR, immunostaining, methylation specific PCR and bisulfate genomic sequencing [9,27]. In this study, hypermethylation of the *TFPI-2 *promoter was detected in 4 of 6 (66.7%) NPC cell lines. We also showed a high frequency (88.6%) of *TFPI-2 *promoter hypermethylation in NPC primary tumor biopsies but not in the NNE tissues, which implied that transcriptional silencing of the TFPI-2 pathway might be involved in NPC tumorigenesis. As well, we found a high frequency of promoter hypermethylation of *TFPI-2 *in early stage (I and II) NPC, which indicates that this might be an early event in NPC carcinogenesis. In contrast to the high frequency of methylation of *TFPI-2 *in NPC cell lines and primary tumors, the absence of promoter hypermethylation in 12 histological NNE tissues showed that hypermethylation of *TFPI-2 *promoter is not necessary to maintain the normal phenotype in the nasopharyngeal epithelium. Thus, hypermethylation of *TFPI-2 *is a frequent and highly tumor-specific event in NPC. Such characteristics may suggest *TFPI-2 *methylation as a molecular marker for NPC.

By bisulfite genomic sequencing assay, we found methylation degrees ranging from 60% to 100% in most of the CpG dinucleotides in the *TFPI-2*-silenced NPC cell lines CNE2 and C666-1. A heavy degree of methylation was also observed in NPC primary tumors, despite the inevitable normal tissue contamination in the biopsy samples without micro-dissection. Moreover, *TFPI-2 *was completely restored by demethylation treatment in all 3 *TFPI-2*-silenced NPC cell lines. In this context, epigenetic inactivation of *TFPI-2 *by promoter hypermethylation is a major, if not the only, mechanism responsible for the loss of *TFPI-2 *expression in NPC.

Although promoter hypermethylation of *TFPI-2 *is frequently found in NPC, it is not associated with sex, age, nodal metastasis or cancer stage in our series. This finding is inconsistent with observations in pancreatic and non-small-cell lung cancer; a high methylation rate of *TFPI-2 *was reported in pancreatic cancer patients with liver metastasis[25]. In non-small-cell lung cancer, *TFPI-2 *promoter hypermethylation was frequently found in patients with late-stage cancer (stages III and IV) and with lymph node metastases[27]. These findings suggest that the prognostic value of promoter hypermethylation of *TFPI-2 *is tissue-specific.

The tumor-suppressive functions of *TFPI-2 *have been demonstrated in several human malignancies such as lung cancer, prostate cancer, glioma, melanoma and esophageal carcinoma. Ectopic expression of TFPI-2 protein in these cancer cell lines negatively regulates colony formation and inhibits cell proliferation[12,27-30]. Additional studies have shown that TFPI-2 inhibits tumor-related angiogenesis and some members of the extracellular matrix (ECM) therefore implicates tumor invasion and progression[8,12,17,29]. Degradation of the ECM is an essential process for tumor invasion and metastasis and involves various matrix-degrading proteinases. The most important proteolytic ECM enzymes are matrix metalloproteinases (MMPs), and upregulated MMP expression has been demonstrated to be strongly associated with the progression of malignancy in several types of cancer, including NPC[31,32]. By inhibiting plasmin, TFPI-2 effectively decreases the activation of MMP-1, MMP-3 and MMP-9 and reduces the invasive potential of several cancer cell lines[9,12,33,34]. In accordance with previous studies, we demonstrated that ectopic expression of *TFPI-2 *significantly inhibited cell proliferation, colony formation and migration in NPC cells. We also demonstrated that restoration of *TFPI-2 *induces apoptosis in NPC cells. All these evidence strongly implies that *TFPI-2 *is a putative TSG and plays a role in nasopharyngeal carcinogenesis.

Methylation-mediated inactivation is reversible, up-regulating *TFPI-2 *by demethylating agent may reverse the malignant phenotype of tumor cells. Therefore TFPI-2 can serve as a novel target for gene therapy in NPC treatment. On the other hand, TFPI-2 functions extracellularly as a secreted protein. Treatment with recombinant TFPI-2 protein inhibited tumor growth and metastasis of esophageal cancer[12]. Thus, TFPI-2 protein might be used directly as an anticancer drug. Further studies will be necessary to explore the great therapeutic potential of *TFPI-2 *in NPC and other cancer.

## Conclusions

The present work demonstrated that epigenetic inactivation of *TFPI-2 *by promoter hypermethylation is a frequent and tumor specific event in NPC and *TFPI-2 *might be considering as a putatitive tumor suppressor gene in NPC.

## Competing interests

The authors declare that they have no competing interests.

## Authors' contributions

SW and XX carried out the molecular studies. CD was responsible for the sample collection and MSP. XZ, YM, JZ performed the sample collection and MSP experiment. LL and XZ was responsible for statistical data analysis. YN and TH carried out the functional studies. GH, NM and MM participated in the design of the study and manuscript review. ZZ conceived of the study, and participated in its design and draft the manuscript. All authors read and approved the final manuscript.

## Pre-publication history

The pre-publication history for this paper can be accessed here:

http://www.biomedcentral.com/1471-2407/10/617/prepub
